# Selections from Surgical Notes

**Published:** 1868-04-01

**Authors:** 


### SELECTION FROM SURGICAL NOTES.

BY PROF. GUNN.

*Case of Excision of Testis, Resection of Humeral Head, and Extirpation of Clavicle.*—In the autumn of 1852, I was consulted by X. Y. Z., on account of an enlargement of one of his testicles, which he attributed to a syphilitic taint acquired the year previous, while sojourning in Paris. The organ was enlarged to twice its natural size, and was rather firmer in texture than its fellow, painless, without preternatural sensitiveness, and inconvenient only from its increased weight. It had undergone the usual treatment by strapping, while mercurials had been duly administered. I put the patient upon the liberal use of *Iodine*, both topical and constitutional. I saw the patient only occasionally during the next eighteen months, during which time the treatment had been but imperfectly carried out; the disease had slowly but steadily increased; his general health had suffered seriously; and my own fears were that the disease was of malignant character. I recommended the removal of the organ; and after making the operation, I put the patient once more upon *Iodine*, on the original idea of the disease — though my fears pointed to a more serious view of the case. Under this treatment, he rapidly regained his health, which he retained until during the winter of 1856, when prolonged exposure to a fierce prairie wind was the exciting cause of violent inflammation and subsequent caries of the left humeral head.

He did not come again under my care until the autumn of 1857, when I resected the diseased portion of the humerus, disarticulating the head of the bone. After the operation, I again put him upon *Iodine*. The wound healed kindly, and his health, which, previous to the operation, had suffered severely, was again rapidly regained.

The patient was cautioned against such exposure as had operated to produce the last development, and instructed as to prompt treatment in case of any manifestation of the disease. Notwithstanding these instructions, he again, in the winter of 1858–9, exposed himself to a fierce and prolonged wind, became thoroughly chilled, and caries of the external extremity of the left clavicle was the result. In October following, I resected the diseased end; but the disease progressed, and in March, 1860, I extirpated the whole bone. In making the operation, I was able to save most of the periosteum, which has reproduced a somewhat irregular, though firm and serviceable bone. After the operation, *Iodine* was again used; and the patient, as on previous occasions, completely regained his flesh, strength and color. During the last attack, he was greatly reduced, and *Iodide of iron* was freely used. Since then, the occasional use of Blanchard's pill of *Iodide of iron* has been necessary — the patient recognizing the indications, and resorting to its use without advice. Under this treatment, he has been enabled to preserve an even, robust condition. The extremity which has lost the humeral head and the whole clavicle, is a very serviceable member. In fact, it is but seldom that the loss would be indicated by the imperfection of his movements.

